# A Novel Mixture Containing *Enterococcus lactis* SF68^®^ Restores Gut Barrier Integrity in a DSS-Induced Murine Model of Post-Colitis IBS-like Symptoms

**DOI:** 10.3390/ijms27156629

**Published:** 2026-07-25

**Authors:** Giulia Valdiserra, Clelia Di Salvo, Letizia Campigli, Vanessa D’Antongiovanni, Carolina Pellegrini, Giada Benedetti, Lara Testai, Cristina Segnani, Laura Benvenuti, Raffaella Coppolecchia, Nunzia Bernardini, Matteo Fornai, Luca Antonioli

**Affiliations:** 1 Unit of Pharmacology and Pharmacovigilance, Department of Clinical and Experimental Medicine, University of Pisa, 56126 Pisa, Italy; giulia.valdiserra@phd.unipi.it (G.V.); clelia.disalvo@phd.unipi.it (C.D.S.); letiziacampigli.pi@gmail.com (L.C.); matteo.fornai@unipi.it (M.F.); luca.antonioli@unipi.it (L.A.); 2 Unit of Histology and Medical Embryology, Department of Clinical and Experimental Medicine, University of Pisa, 56126 Pisa, Italy; 3 Department of Pharmacy, University of Pisa, 56126 Pisa, Italylara.testai@unipi.it (L.T.); 4 Unit of Experimental Biology and Genetics, Department of Clinical and Experimental Medicine, University of Pisa, 56126 Pisa, Italy; 5 Cerbios-Pharma SA, 6917 Lugano, Switzerland

**Keywords:** irritable bowel syndrome, *Enterococcus lactis* SF68^®^, probiotics, gut, inflammation, butyrate

## Abstract

(1) Irritable bowel syndrome (IBS) is a functional gastrointestinal disorder characterized by abdominal pain and altered bowel habits, linked to dysbiosis and inflammation. Currently available treatments, focused on symptom management, displayed limited efficacy. Recent research highlights microbiota modulation as a potential therapeutic strategy to restore intestinal barrier integrity; (2) A dextran sulfate sodium (DSS)-induced mouse model of post-colitis IBS-like symptoms was performed to evaluate the effect of a novel mixture of *Enterococcus lactis* SF68^®^, butyrate, and folate. Disease activity index and spleen weight were assessed. Markers of gut inflammation, intestinal barrier alterations, and gut butyrate bioavailability were measured. The activity of colonic mitochondria was also analyzed; (3) Supplementation with the novel mixture significantly reduced spleen weight and cytokine levels. The intestinal epithelial barrier was restored through an increase in claudin-1, occludin, and defensin 1–3 and a reduction in LBP plasma level. Treatment with the mixture enhanced the expression of butyrate transporters in mice. Mitochondrial activity markers, including citrate synthase and Cytochrome *c* oxidase, were improved by treatment; (4) The novel mixture containing *Enterococcus lactis,* butyrate, and folate exerted a protective effect against post-inflammatory IBS by reducing local inflammation, restoring intestinal barrier integrity, and enhancing butyrate bioavailability, suggesting its potential for managing post-inflammatory IBS symptoms effectively.

## 1. Introduction

Irritable bowel syndrome (IBS) is a functional gastrointestinal disease characterized by chronic recurrent abdominal pain, discomfort, changes in defecation habits, and abnormal stool form [1]. IBS affects 12% of the global population, and it is associated with high comorbidity with psychological disorders as well as a relevant disease burden with regard to a decrease in work productivity [2].

Despite the pathophysiology of IBS remaining partially understood, this syndrome displays alterations in the gut microbiome (dysbiosis), which can promote the development of chronic, low-grade, subclinical inflammation leading to an increase in intestinal permeability, pivotally involved in the development and progression of the disease [3].

Nowadays, the pharmacological approaches for IBS are aimed at targeting symptoms. In this regard, the main drugs used are antispasmodics and loperamide to manage abdominal pain and to reduce bowel contractions, respectively [4]. Contextually, a diet regimen with low fermentable small-chain carbohydrates is strictly recommended in all IBS patients, to reduce the gas production and intestinal osmolality [4]. The scarce efficacy and/or safety of traditional drugs and the large heterogeneity of clinical manifestations of the disease lead researchers to identify novel strategies to improve the management of IBS.

Recent studies showed differences in terms of gut microbiota composition in IBS patients compared to healthy people. *Bifidobacterium*, *Lactobacillus*, and *Faecalibacterium prausnitzii* were decreased in IBS patients, while an increase in *Firmicutes*, *Clostridia*, and *Escherichia coli* was reported. In this regard, increasing attention has been focused on the manipulation of gut microbiota, via pre- and probiotics, as a complementary approach in addition to the traditional pharmacological tools [3]. However, available data regarding the supplementation with pre- and probiotics are contradictory.

Several studies demonstrated that probiotic supplementation may be useful in the treatment of IBS (VSL#3, *Lactobacillus*, *Bifidobacterium*) [5,6], restoring the intestinal permeability through the production of anti-inflammatory cytokines. By contrast, other evidence showed that the above-mentioned bacterial strains worsened IBS symptoms, increasing the abdominal pain and flatulence [5].

In recent years, there has been an increasing interest also in the effect of short-chain fatty acids (SCFA), microbiota metabolites, on the intestinal epithelial cells [7]. In particular, butyrate displays an anti-inflammatory activity by interacting with G-protein coupled receptors (GPCRs) in the intestinal mucosa. However, recent evidence showed that butyrate does not protect against barrier dysfunction in patients with intestinal inflammation [8].

It is emerging that patients with chronic gut inflammation (i.e., IBS, ulcerative colitis, and Crohn’s disease) are characterized by an increased fecal concentration of butyrate, due to a reduction in SCFA transporter monocarboxylate transporter 1 (MCT1) expression [8,9]. Pre-clinical and clinical studies highlighted that the supplementation with *Enterococcus lactis* SF68^®^ reduced the intestinal inflammation, ameliorating the bioavailability of butyrate at the mucosal level, via the restoration of the colonic expression of butyrate apical transporter SMCT1 [10].

Based on these premises, the present study aimed to evaluate the ability of co-administration of SF68 butyrate and folate in counteracting the immune–inflammatory and epithelial barrier dysfunction in a murine model of post-colitis IBS-like symptoms.

## 2. Results

### 2.1. Evaluation of the Disease Activity Index and Macroscopic Parameters

**Figure 1:**
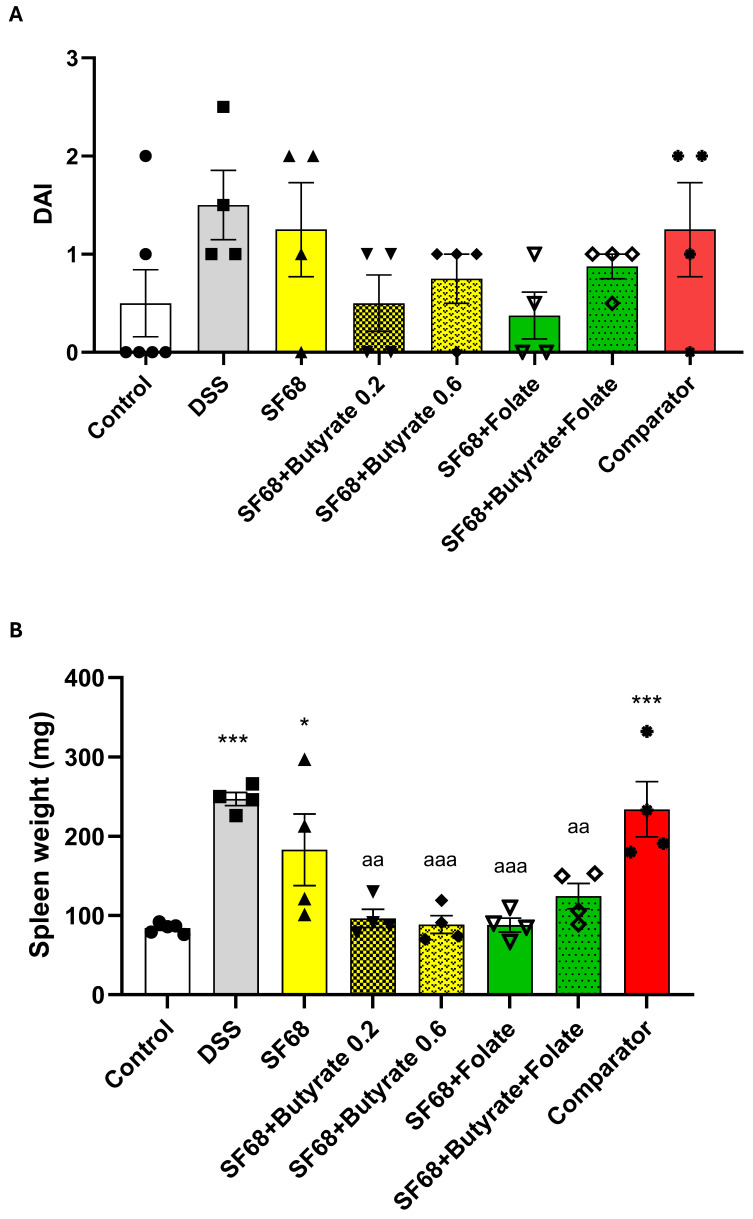
(**A**) DAI and (**B**) spleen weight in control mice or DSS mice treated with vehicle or SF68, SF68 + Butyrate 0.2 mg/kg, SF68 + Butyrate 0.6 mg/kg, SF68 + Folate, SF68 + Butyrate + Folate, Comparator. Data are expressed as mean ± S.E.M. of 4–6 animals. Statistics: One-way ANOVA followed by Tukey post-hoc test. * *p* < 0.05 vs. Control, *** *p* < 0.001 vs. Control; ^aa^ *p* < 0.01 vs. DSS; ^aaa^ *p* < 0.001 vs. DSS.

On the day of sacrifice, DSS mice showed a slight increment of the DAI and a significant increment of spleen weight compared to the control group (Figure 1A,B). The treatment with SF68 alone or in combination with folate and/or butyrate reduced the spleen weight but not the DAI (Figure 1A,B). No effect was observed in mice with post-colitis IBS administered with a comparator for both parameters.

### 2.2. Plasma Levels of LBP

**Figure 2:**
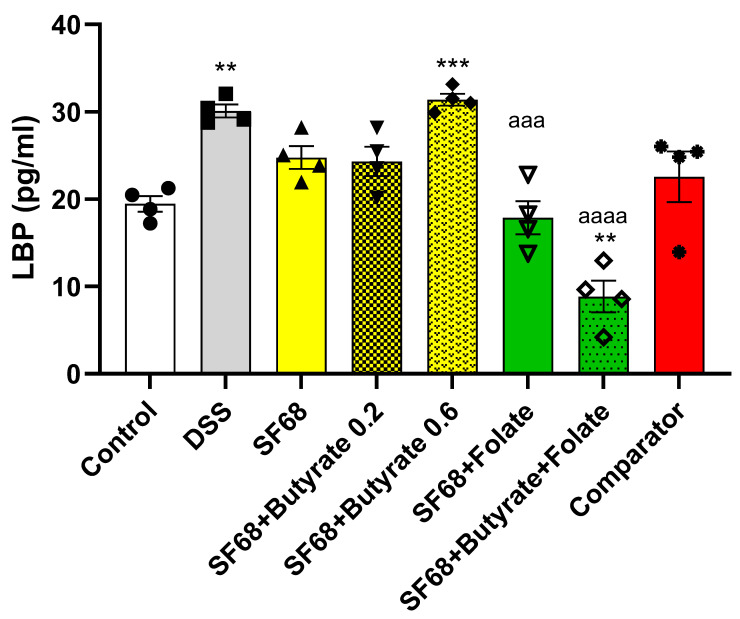
Plasma levels of LBP in control mice or DSS mice treated with vehicle or SF68, SF68 + butyrate 0.2 mg/kg, SF68 + butyrate 0.6 mg/kg, SF68 + Folate, SF68 + Butyrate + Folate, Comparator. Data are expressed as mean ± S.E.M. of 4–6 animals. Statistics: One-way ANOVA followed by Tukey post-hoc test. ** *p* < 0.01 vs. Control, *** *p* < 0.001 vs. Control; ^aaa^ *p* < 0.001 vs. DSS; ^aaaa^ *p* < 0.0001 vs. DSS.

The LBP plasma levels, a marker of intestinal permeability, were significantly increased in the DSS group compared to the control. Of note, DSS mice treated with SF68, plus butyrate and folate, showed a significant decrease compared to DSS mice (Figure 2). On the other hand, no effect was observed in DSS-mice administered with the comparator (Figure 2).

### 2.3. Analysis of Colonic Interleukin IL-1β, IL-6, IL-10 and Tumor Necrosis Factor (TNF)

**Figure 3:**
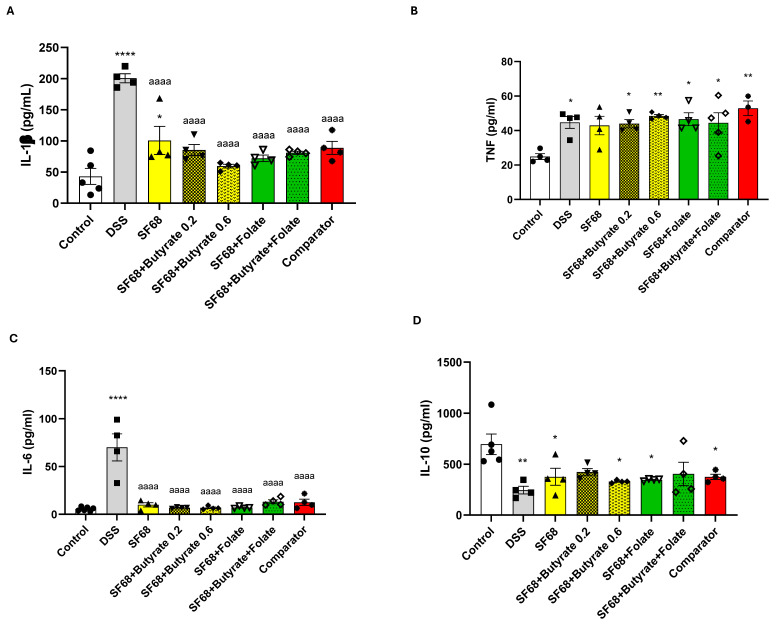
Colonic levels of (**A**) IL-1β, (**B**) TNF, (**C**) IL-6, and (**D**) IL-10 in control mice or DSS mice treated with vehicle or SF68, SF68 + butyrate 0.2 mg/kg, SF68 + butyrate 0.6 mg/kg, SF68 + Folate, SF68 + Butyrate + Folate, Comparator. Data are expressed as mean ± S.E.M. of 4–5 animals. Statistics: One-way ANOVA followed by Tukey post-hoc test. * *p* < 0.05 vs. Control, ** *p* < 0.01 vs. Control, **** *p* < 0.0001 vs. Control; ^aaaa^ *p* < 0.0001 vs. DSS.

Colonic damage induced by DSS was associated with an increase in pro-inflammatory cytokines as well as a reduction in anti-inflammatory cytokines. In line with this view, the mice with post-inflammatory IBS displayed significant increases in IL-1β, IL-6, and TNF and a significant reduction in IL-10 in comparison with control animals (Figure 3A,C,D). The tissue levels of IL-1β and IL-6 were significantly reduced by all the test drugs in DSS mice compared to untreated DSS animals, but no effect on the IL-10 colonic level. By contrast, colonic TNF levels were not altered in all experimental groups compared to DSS (Figure 3B).

### 2.4. Western Blot Analysis of Tight Junction Proteins

**Figure 4:**
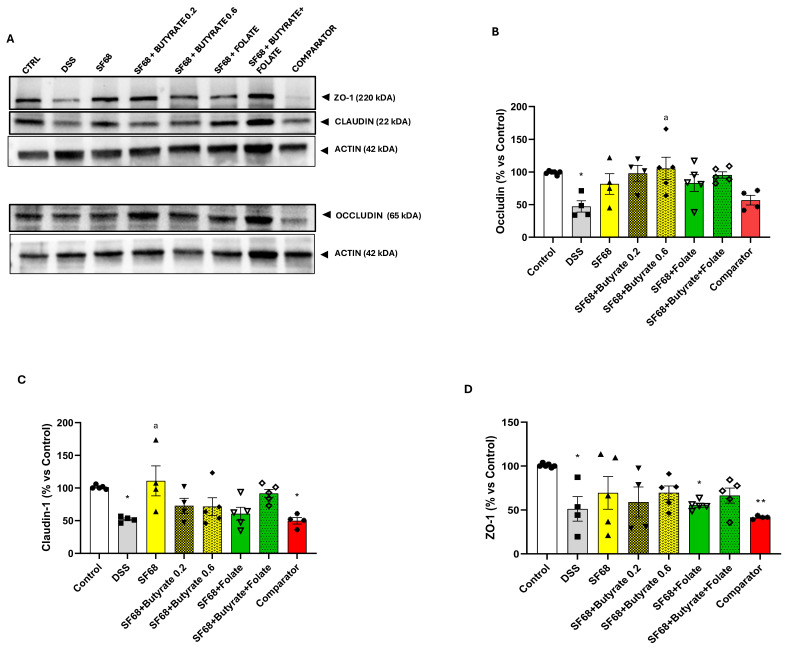
(**A**) Representative image and densitometric analysis of (**B**) occludin, (**C**) claudin-1, and (**D**) ZO-1 expression levels in colonic tissues from control mice or DSS mice treated with vehicle or SF68, SF68 + butyrate 0.2 mg/kg, SF68 + butyrate 0.6 mg/kg, SF68 + Folate, SF68 + Butyrate + Folate, Comparator. Data are expressed as mean ± S.E.M. of 4–5 animals. Statistics: One-way ANOVA followed by Tukey post-hoc test. * *p* < 0.05 vs. Control, ** *p* < 0.01 vs. Control; ^a^ *p* < 0.05 vs. DSS.

The integrity of tight junction proteins is often compromised in the presence of gut inflammation, resulting in increased intestinal permeability. In line with the data shown in Figure 2, DSS-treated mice exhibited a significant reduction in occludin, claudin-1, and ZO-1 expression compared with the control group (Figure 4A–D). Treatment with SF68 combined with butyrate at 0.6 mg/kg significantly improved occludin expression (Figure 4B), whereas SF68 alone restored claudin-1 levels (Figure 4C). Notably, the administration of SF68 plus butyrate at 0.2 mg/kg and folate normalized both occludin and claudin-1 expression (Figure 4B,C). No changes in occludin or claudin-1 were observed following treatment with the comparator (Figure 4B,C). Finally, DSS administration markedly reduced ZO-1 levels, but none of the tested treatments elicited significant effects on its expression (Figure 4D).

### 2.5. Western Blot Analysis of Butyrate Transporters

Short-chain fatty acids (SCFA), such as butyrate and propionate, produced by gut microbiota, may permeate through the cell membrane by passive diffusion, but mainly SCFA are absorbed via specific transporters, such as MCT1 (apical), MCT4 (basolateral), and SMCT1 (apical). It is emerging that chronic gut inflammation is characterized by a reduced expression of these transporters with a decrease in SCFA absorption [11].

**Figure 5:**
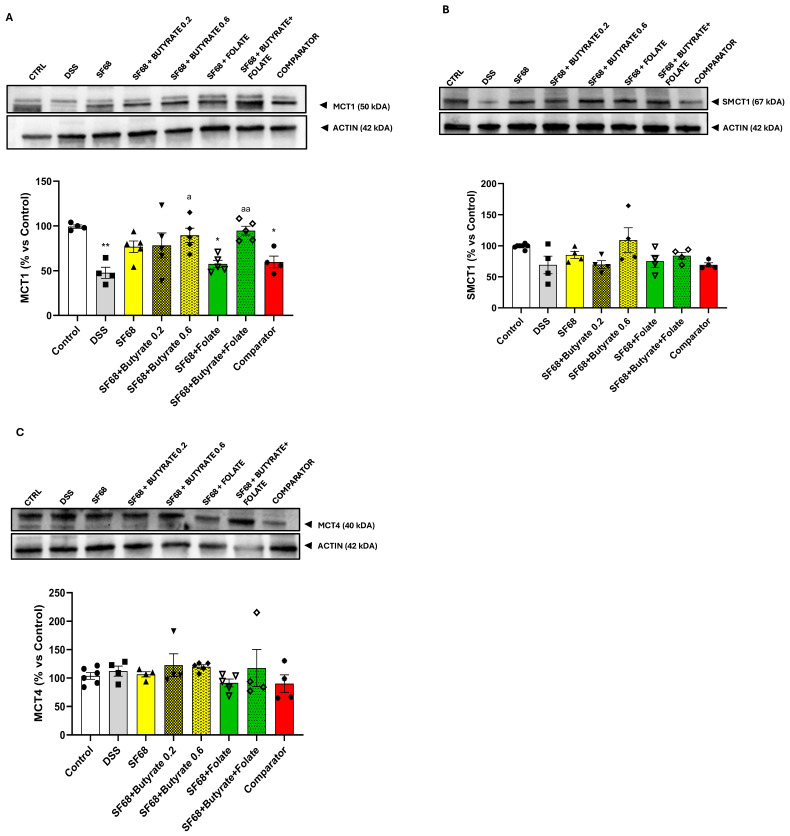
(**A**–**C**) Representative image and densitometric analysis of (**A**) MCT1, (**B**) SMCT1, and (**C**) MCT4 expression levels in colonic tissues from control mice or DSS mice treated with vehicle or SF68, SF68 + butyrate 0.2 mg/kg, SF68 + butyrate 0.6 mg/kg, SF68 + Folate, SF68 + Butyrate + Folate, Comparator. Data are expressed as mean ± S.E.M. of 4–6 animals. Statistics: One-way ANOVA followed by Tukey post-hoc test. * *p* < 0.05 vs. Control, ** *p* < 0.01 vs. Control; ^a^ *p* < 0.05 vs. DSS; ^aa^ *p* < 0.01 vs. DSS.

In line with this evidence, DSS mice showed a significant reduction in the butyrate transporter MCT1 compared to the control group. SF68 + butyrate 0.6 mg/kg, as well as SF68+butyrate 0.2 mg/kg + folate, but not comparator administration, provoked a significant restoration of MCT1 expression (Figure 5A,B). By contrast, treatment with DSS or with all test drugs did not affect SMCT1 or MCT4 expression levels (Figure 5C).

### 2.6. Citrate Synthase and Cytochrome C Oxidase Activities

**Figure 6:**
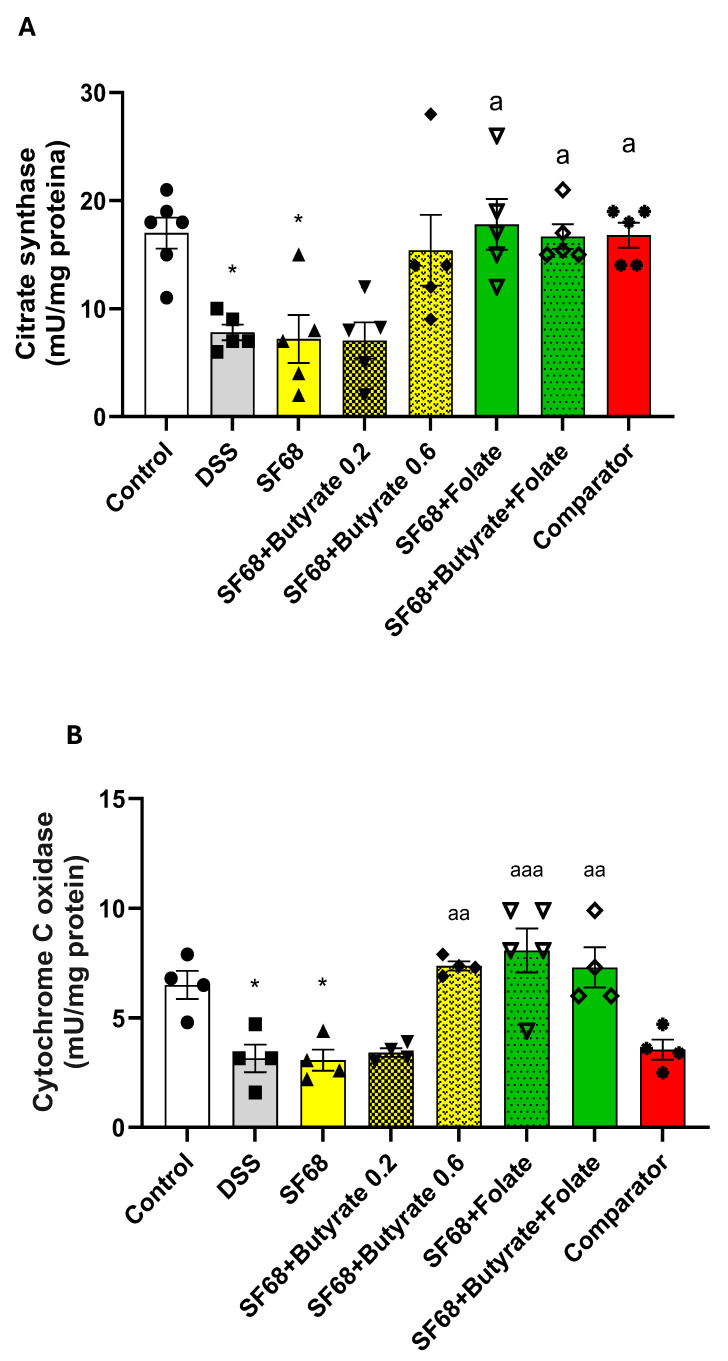
(**A**) Citrate synthase and (**B**) Cytochrome *c* oxidase activity in colonic tissues from control mice or DSS mice treated with vehicle or SF68, SF68 + butyrate 0.2 mg/kg, SF68 + butyrate 0.6 mg/kg, SF68+Folate, SF68 + Butyrate + Folate, Comparator. Data are expressed as mean ± S.E.M. of 4–6 animals. Statistics: One-way ANOVA followed by Tukey post-hoc test. * *p* < 0.05 vs. Control; ^a^ *p* < 0.05 vs. DSS; ^aa^ *p* < 0.01 vs. DSS; ^aaa^ *p* < 0.001 vs. DSS.

The citrate synthase and cytochrome *c* oxidase are both parameters of mitochondrial activity [12]. Impairments in mitochondrial respiration are hallmarks of several diseases, including IBDs, and alterations in citrate synthase levels are considered early markers of intestinal epithelial dysfunction [13]. In this context, DSS-induced post-colitis IBS-like model caused a significant reduction in both citrate synthase (Figure 6A) and cytochrome *c* oxidase (Figure 6B) activity compared with controls. Supplementation with SF68 plus folate or with SF68 combined with butyrate at 0.2 mg/kg and folate, as well as treatment with the comparator, significantly restored citrate synthase activity (Figure 6A). Moreover, DSS mice receiving SF68 plus butyrate at 0.6 mg/kg, SF68 plus folate, or SF68 combined with butyrate at 0.2 mg/kg and folate exhibited a marked increase in cytochrome *c* oxidase activity compared with post-colitis IBS animals (Figure 6B). In contrast, the comparator treatment did not affect cytochrome *c* oxidase activity in DSS mice (Figure 6B).

### 2.7. Expression and Distribution of Paneth Cell-Released Defensins in the Ileum

**Figure 7:**
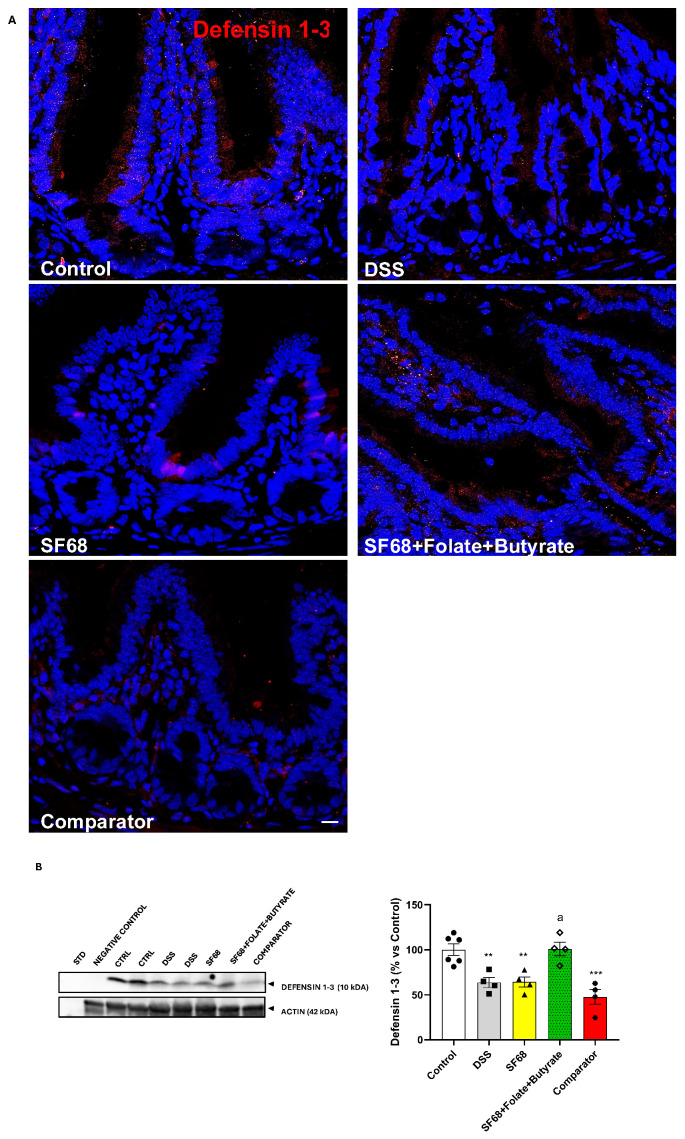
(**A**) Representative photomicrographs of Defensin 1–3 immunostaining in cross-sectioned ileum from control and DSS mice treated with vehicle or SF68, SF68 + Butyrate + Folate, or comparator. Original magnification 63×. Scale bar: 10 µm. The images are shown for selected experimental groups to illustrate the main treatment effects. (**B**) Densitometric analysis of Defensin 1–3 in ileal tissues from control and DSS mice treated with vehicle or SF68, SF68 + Butyrate + Folate, Comparator. Data are expressed as mean ± S.E.M. of 4–6 animals. Statistics: One-way ANOVA followed by Tukey post-hoc test. ** *p* < 0.01 vs. Control, *** *p* < 0.001 vs. Control, ^a^ *p* < 0.05 vs. DSS.

In the intestinal epithelium, Paneth cells, residing at the base of the crypts of Lieberkühn, release secretory granules rich in α-defensins (1–3), regarded as cryptdins (Crps) in mice and HD5 and HD6 in humans, in response to bacteria and other stimuli, thus contributing to intestinal innate immunity [14]. In addition, Paneth cell-released α-defensins modulate gut microbiota composition, intestinal barrier integrity, and affect host-adaptive immunity [12]. Disturbances in Paneth cell function, arising from inflammatory stimuli, genetic susceptibility, or age-related alterations, can impair antimicrobial peptide production, leading to microbial dysbiosis as well as compromised barrier integrity and thereby, promoting intestinal and systemic inflammation [15,16]. In this context, Western blot analysis showed that DSS-treated mice displayed a significant decrease in ileal defensin 1–3 expression as compared with control animals (Figure 7B). The combination of SF68 plus butyrate and folate normalized defensin 1–3 expression (Figure 7B). Immunofluorescence analysis of ileal sections showed defensin 1–3 immunopositivity in intestinal crypts and lumen from control animals as compared with DSS mice. Of note, DSS animals treated with SF68 plus butyrate and folate displayed an increased defensin 1–3 distribution in the intestinal crypts and lumen as compared with the DSS group (Figure 7A).

## 3. Discussion

The present study demonstrates that supplementation with *Enterococcus lactis* SF68^®^, butyrate, and folate exerts a multifaceted protective effect in a DSS-induced post-colitis IBS-like model, reducing intestinal inflammation, restoring epithelial barrier integrity, and improving mitochondrial function.

We found that the administration of this novel mixture exerted a multifaceted protective effect in the DSS-induced post-colitis IBS-like model. In particular, it mitigated colonic inflammation by reducing tissue levels of the proinflammatory cytokines IL-1β and IL-6, thereby attenuating the local immune response. In parallel, the supplement restored the intestinal epithelial barrier (IEB) integrity, a key determinant of gut homeostasis and resilience to recurrent injury. Moreover, it supported butyrate bioavailability within the colonic epithelium, which was associated with a consequent improvement in mitochondrial activity, highlighting a pivotal role in sustaining cellular energy metabolism and barrier function.

The present study was performed using a DSS-mediated post-colitis IBS-like model, since it reproduces the sequence of acute colitis followed by a phase of persistent visceral hypersensitivity in the absence of overt inflammation [16]. In this model, oral DSS administration damages the colonic epithelium, triggering an acute inflammatory response that gradually recovers after DSS withdrawal [16]. Despite apparent histological recovery, animals develop long-lasting alterations in gut function, including enhanced pain perception to colorectal distension, increased intestinal permeability, and changes in enteric and immune signaling [17]. These hallmarks closely resemble the post-inflammatory state observed in patients who develop IBS following infectious or inflammatory colitis, underscoring the DSS model as a valuable tool for elucidating disease mechanisms and assessing potential therapeutic interventions [18].

In this murine model of post-colitis IBS, the supplementation with SF68^®^, butyrate, and folate, either alone or in combination, tended to reduce the spleen weight and the DAI score, suggesting a beneficial effect on disease severity. To further support the anti-inflammatory effect of SF68 alone or in combination with butyrate and/or folate, we measured the tissue levels of both pro- and anti-inflammatory cytokines. DSS administration led to increased levels of IL-1β and IL-6, consistent with clinical evidence supporting the central role of these cytokines in driving gut barrier dysfunctions [19]. Indeed, IL-1β and IL-6 play a central role in disrupting the integrity of the intestinal epithelial barrier by targeting tight junctions (TJs), which are essential for regulating paracellular permeability. IL-1β activates intracellular signaling pathways, including NF-κB and MAPKs, increasing the expression of myosin light chain kinase (MLCK). MLCK phosphorylates myosin light chains, promoting actomyosin contraction, which disrupts the structure and localization of key TJ proteins such as occludin, claudins, and ZO-1, thereby increasing intestinal permeability. On the other hand, IL-6 binds to its receptor and signals through the gp130 co-receptor, activating the MEK/ERK and PI3K/Akt pathways. This promotes transcriptional upregulation of claudin-2, a pore-forming TJ protein that selectively increases paracellular permeability to cations. Together, these alterations compromise barrier function and facilitate bacterial translocation [20,21].

Treatment with SF68 and/or microbiota-derived metabolites reduced the level of pro-inflammatory cytokines, supporting the concept that targeting the gut microbiota can be a therapeutic strategy to mitigate intestinal inflammation and alleviate IBS symptoms [22,23]. In our post-colitis IBS-like model, the pro-inflammatory cytokine TNF only slightly increased. Notably, clinical studies have reported variable TNF levels in IBS patients, depending on disease subtype, genetic polymorphisms, sample source (serum vs. mucosal biopsies), and anatomical site of analysis. Moreover, anti-TNF therapies, which are highly effective in IBD, have shown no clear benefit in IBS, further supporting the notion that TNF is not a central mediator in IBS pathophysiology [24,25,26,27]. Supplementation with SF68^®^, butyrate, and folate did not significantly affect TNF concentrations in this model. The modest modulation of TNF observed in our post-colitis IBS-like model is consistent with clinical evidence and does not detract from the relevance of targeting alternative inflammatory and barrier-related pathways in IBS.

As previously discussed, IBS symptoms arise from a complex interplay between immunity, dysbiosis, and inflammation, which collectively alter the epithelial barrier integrity [28]. In detail, patients with IBS exhibit enhanced colonic permeability (leaky gut), allowing luminal antigens and microbial products to translocate into the submucosa and systemic circulation [29,30,31]. In line with this view, our post-inflammatory IBS model showed a reduction in all tight junctions, key regulators of intestinal epithelium integrity, contributing to barrier dysfunction. This impairment was further confirmed by an increased plasma LBP level.

Administration of SF68^®^ alone, in combination with butyrate, or as part of the full mixture restored occludin and claudin expression. These findings are in line with other evidence demonstrating the ability of SF68^®^ ([10] and butyrate to enhance epithelial barrier integrity [32,33]. Moreover, folate has been reported to strengthen the IEB, acting directly on tight junction expression as well as shaping microbiota composition toward “protective” bacteria [34,35]. In line with these findings, plasma LBP levels were reduced in the mixture-treated group, further supporting the restoration and reinforcement of gut barrier function.

It is well known that gut microbiota, producing SCFAs, participate actively in maintaining gastrointestinal barrier function [36]. Among SCFAs, butyrate is absorbed in the colon primarily via specific membrane transporters, notably the proton-coupled monocarboxylate transporter 1 (MCT1) and the sodium-coupled monocarboxylate transporter 1 (SMCT1). These transporters mediate butyrate uptake from the colonic lumen into epithelial cells, where butyrate serves as a key energy substrate. MCT1 expression is upregulated by butyrate itself, enhancing its intracellular availability [7,37]. It is worth noting that gut inflammatory conditions, including IBDs or IBS, are associated with a reduced expression of butyrate transporters [7,38,39,40]. A similar decrease was observed in a mouse model of diet-induced obesity characterized by mild intestinal inflammation [10]. In this context, our group previously demonstrated that SF68 can modulate the expression of SMCT1, thereby facilitating luminal butyrate absorption [10]. Building on these findings, we also investigated the effects of the probiotic and its metabolites on MCT1, SMCT1, and MCT4. Interestingly, treatment with SF68^®^ combined with butyrate, as well as with the full mixture, led to a significant increase in MCT1 expression [41,42,43]. While in our previous study the probiotic primarily modulated SMCT1 expression, the present results indicate a preferential effect on MCT1. This apparent difference may reflect the context-dependent regulation of SCFA transporters, which can be influenced by the specific inflammatory pathways involved, the disease model, tissue metabolic status, and treatment conditions. In particular, MCT1 expression is highly responsive to inflammatory and metabolic alterations associated with epithelial injury and tissue repair, which may explain its selective modulation in the current experimental setting [44]. These results further confirm the ability of SF68^®^ and butyrate to restore proper SCFA absorption and utilization by enterocytes [41,42,43].

Butyrate is widely recognized for its fundamental role in modulating the transcription of genes involved in key metabolic pathways, including pyruvate dehydrogenase, the tricarboxylic acid (TCA) cycle, and the mitochondrial respiratory chain. Among the TCA enzymes, citrate synthase catalyzes the condensation of oxaloacetate and acetyl-CoA to form citrate, while cytochrome *c* oxidase facilitates the transfer of electrons from cytochrome *c* to oxygen, producing water and driving ATP synthesis. Assessing the activity of these enzymes provides a measure of mitochondrial respiratory function before and after supplementation with the test compounds [37,45]. Of note, animals treated with our novel mixture, but not with the comparator, exhibited improved mitochondrial respiration, as evidenced by increased activity of citrate synthase and cytochrome *c* oxidase.

In this context, insufficient levels of butyrate have been shown to impair Paneth cell activity, leading to a reduced secretion of key antimicrobial peptides, such as defensins 1–3 [46]. These peptides are essential for maintaining intestinal barrier integrity and modulating the mucosal immune response. Dysregulation of Paneth cells may therefore contribute to increased IEB permeability, promoting a pro-inflammatory microenvironment [47]. Consistent with these findings, treatment with a mixture containing SF68, butyrate, and folate significantly increased ileal expression levels of defensins 1–3, highlighting the ability of this combination to restore Paneth cell function and enhance gut barrier homeostasis. When considering the effect of the comparator, containing *Bifidobacterium*, butyrate, and fructo-oligosaccharides, we observed limited efficacy.

This study has some limitations that should be acknowledged. First, although the DSS-induced post-colitis model is widely used to investigate features associated with post-inflammatory IBS, visceral hypersensitivity, a hallmark of IBS, was not directly assessed in the present study. Therefore, the extent to which the model fully reproduces all aspects of IBS pathology cannot be established. Second, the study was primarily designed to evaluate the efficacy of the tested mixture; consequently, treatment groups receiving butyrate or folate alone were not included. While this approach allowed us to focus on the effects of the formulation and to reduce animal use in accordance with ethical considerations, the individual contribution of each component cannot be fully determined. Third, the relatively small sample size, the absence of a priori sample size calculation, and the lack of blinding represent additional limitations that may affect the robustness of the findings. Finally, although the statistical approach employed is commonly used in similar experimental studies, the limited number of animals per group should be considered when interpreting the results. Therefore, further studies including larger cohorts, additional control groups, and functional assessments of visceral sensitivity are warranted to confirm and extend the present findings.

Indeed, this supplement, despite ameliorating specific parameters, such as DAI, spleen weight, levels of tissue cytokines, and citrate synthase, displayed scarce efficacy on LBP, tight junctions, butyrate transporter MCT1, cytochrome *c* oxidase, and defensin 1–3. Based on this evidence, it is conceivable that the comparator exerted a beneficial activity mainly counteracting intestinal inflammation but had a negligible effect on the amelioration of intestinal epithelial barrier. Indeed, it failed to restore the tight junction expression, thus allowing an influx of LBP into the plasma. The limited impact on intestinal mucosa may result from inadequate regulation of butyrate uptake and metabolism, as evidenced by the unchanged levels of MCT1 and cytochrome *c* oxidase.

## 4. Materials and Methods

### 4.1. Animals

Male C57BL/6 mice, five-week-old, were obtained from ENVIGO S.r.l (San Pietro al Natisone, UD, Italy) and were used throughout the study. The animals, after arriving at the laboratory, were randomly housed for one week in the absence of experimental procedure. Mice were fed with standard laboratory chow and tap water ad libitum. They were housed, six mice in a cage, in temperature-controlled rooms with a 12 h light cycle at 22–24 °C and 50–60% humidity. All the procedures involving animals were performed following the guidelines of the European Community Council Directive 2010/63/UE and in accordance with the Declaration of Helsinki, EU Directive 2010/63/EU for animal experiments. All the experiments have been approved by the Ethical Committee for Animal Experimentation of the University of Pisa and by the Italian Ministry of Health (Authorization No. 176/2022-PR, 9 March 2022). All efforts were made to reduce and minimize the number of animals and the suffering, respectively.

### 4.2. Experimental Design

The induction of colitis followed the methods described previously [48]. In detail, mice received 2.5% of DSS [molecular weight 40 kDa (*w*/*v*)] for 5 days and normal drinking water for three weeks [16]. All groups of animals had similar daily fluid intake. Food intake has been monitored daily starting from the onset of experimental protocols. In addition, mice have been weighed each morning for the duration of the experiments, and they have been examined for diarrhea and the presence of blood in the stool or rectal bleeding.

Mice were randomly housed in eight separate experimental groups of 6 mice each. After 2 weeks of recovery, animals were administered once daily via oral gavage for 1 week with the test drugs (Figure S1). Carboxymethyl cellulose (0.3%) was utilized as a vehicle for all groups.

The treatment groups were arranged as follows:

- *Enterococcus lactis* SF68^®^ (SF68, 1 × 10^8^ CFU/mice)
- *Enterococcus lactis* SF68^®^ + butyrate 0.20 mg/kg/day (low dose)
- *Enterococcus lactis* SF68^®^ + butyrate 0.60 mg/kg/day (normal dose)
- *Enterococcus lactis* SF68^®^ + folate
- *Enterococcus lactis* SF68^®^ + butyrate low-dose butyrate + folate
- *Bifidobacterium lactis* (1 × 10^9^ CFU) *Bifidobacterium lactis* (1 × 10^9^ CFU), calcium butyrate 250 mg, and fructo-oligosaccharides 100 mg (4.3 mg/mice/day, comparator, product commercially available)

At the end of the experimental protocol, animals were euthanized to collect blood samples, feces, spleen, and colonic tissues.

### 4.3. Assessment of the Disease Activity Index

At the time of sacrifice, the disease activity index (DAI) was calculated by assigning scores to changes in body weight (0: <1%; 1: 1–5%; 2: 5–10%; 3: 10–15%; 4: >15%), rectal bleeding (0: negative; 2: presence of blood in the stool; and 4: gross rectal bleeding) and stool consistency (0: normal; 2: soft; and 4: diarrhea) [49].

### 4.4. Evaluation of Plasma Levels of Lipopolysaccharide-Binding Protein (LBP)

Lipopolysaccharide-binding protein (LBP) levels in plasma were measured by enzyme-linked immunosorbent assay (ELISA) kits (Abcam, Cambridge, UK, ab213876 LBP) as described by the manufacturer’s instructions. For this purpose, blood samples were centrifuged for 5 min at 4000× *g* at 2–8 °C. Supernatants were collected, and aliquots (100 μL) of plasma were used to perform the assay. The results were expressed as picogram per milliliter (ρg/mL).

### 4.5. Evaluation of Colonic Interleukin (IL)-1β, IL-6, IL-10 and Tumor Necrosis Factor (TNF)

The levels of IL-1β, IL-6, IL-10, and TNF in colonic tissues were measured by ELISA (Invitrogen, Carlsbad, CA, USA), as described by the manufacturer’s instructions. Tissue samples from animals, previously stored at −80 °C, were weighed, thawed, and homogenized in 0.3 mL of PBS (pH 7.2) at 4 °C and centrifuged at 12,000× *g* for 30 min. Aliquots (100 μL) of supernatants were used to perform the assay. The results were expressed as picogram per milligram of tissue protein (ρg/mg).

### 4.6. Protein Extraction and Western Blot Analysis

Colonic tissues were weighed and homogenized in lysis buffer, using a polytron homogenizer (QIAGEN, Milan, Italy). Then, samples were sonicated and boiled for 5 min at 95 °C. The Bradford assay was used to quantify protein concentrations. Proteins were separated onto a pre-cast 4–20% Criterion™ TGX™ Precast Midi Protein Gel (Bio-Rad, Hercules, CA, USA) and transferred to PVDF membranes (Trans-Blot Turbo^TM^ PVDF Transfer packs, Bio-Rad, CA, USA). Membranes were blocked with 3% bovine serum albumin (BSA) diluted in Tris-buffered saline (TBS, 20 mM Tris-HCl, PH 7.5, 150 mM NaCl) with 0.1% Tween20. Primary antibodies against monocarboxylate transporter 1 (MCT1 PA5-76687), monocarboxylate transporter 4 (MCT4 PA5-106683), sodium-coupled monocarboxylate transporter 1 (SMCT1, BS-6106R), occludin (ab167161), zonulin-1 (ab96587), claudin 1 (ab180158), β-actin (ab8227), and glyceraldehyde-3-phosphate dehydrogenase (GAPDH, ab8245), defensin 1–3 (PA5103122) were used. Secondary antibodies were obtained from Abcam (anti-mouse ab97040 and anti-rabbit ab6721). Protein bands were detected with enhanced chemiluminescence (ECL) reagents (Clarity Western ECL Blotting Substrate, BioRad, CA, USA). Densitometry was performed by IBright Analysis 5.6.0 software (Invitrogen, Thermo Fisher Scientific, Carlsbad, CA, USA).

### 4.7. Evaluation of Citrate Synthase Activity and Cytochrome C Oxidase Activity on Ileum

Ileum tissue was weighed and added to a solution containing 500 μL of STE at pH 7.4 and 10 μL of Triton X-100 (0.02% *v*/*v*) (T9284, Sigma-Aldrich, St. Louis, MO, USA). STE was composed of sucrose (250 mM, 84100, Sigma-Aldrich, St. Louis, MO, USA), EGTA (1 mM, E4378, Sigma-Aldrich, St. Louis, MO, USA), and Tris (5 mM, T4661, Sigma-Aldrich, St. Louis, MO, USA). The mixture was kept constantly on ice at 4 °C and then homogenized with a GentleMACS dissociator (Miltenyi Biotec, Bergisch Gladbach, Germany). The tissue homogenate obtained was transferred to an Eppendorf tube and centrifuged for 15 min at 12,000× *g* and 4 °C. After centrifugation, the supernatant was removed and transferred to another Eppendorf tube, kept on ice for the duration of the experiment, while the pellet was discarded.

The Citrate synthase activity was evaluated on samples diluted in Tris-buffer (100 mM; pH 8.2) after the addition of a solution containing 5,5′-dithiobis-2-nitrobenzoic acid (DTNB, 100 μM) (D8130, Sigma-Aldrich, St. Louis, MO, USA) and acetyl-coenzyme A (100 μM) (A2056, Sigma-Aldrich, St. Louis, MO, USA). The assay was performed in 96 multi-well plates (1 μg of proteins per well) and the reaction was started by the addition of a solution of oxaloacetic acid (500 μM) (O4126, Sigma-Aldrich, St. Louis, MO, USA) The reaction was followed spectrophotometrically at 37 °C every 30 s for 15 min at the wavelength of 412 nm (EnSpire, PerkinElmer, Waltham, MA, USA). Citrate synthase activity was determined by comparing the sample’s activity with a known concentration of the isolated enzyme (C3260-200UN, Sigma-Aldrich, St. Louis, MO, USA). Citrate synthase activity is expressed in mU/mg of protein.

As concerns the Cytochrome *c* oxidase activity, it was monitored by using a colorimetric assay. As the first step, the reduction of Cytochrome *c* (C2037, Sigma-Aldrich, St. Louis, MO, USA) was carried out by adding 5 μL of 10^−1^ M DTT into an Eppendorf containing exactly 1 mL of Cytochrome *c* for 15 min at room temperature until the solution changed color from dark red to light purple. To verify the reduction of Cytochrome *c*, a spectrophotometric measurement at 550 and 565 nm was carried out, and the ratio between the absorbances was then measured. The reduction of Cytochrome *c* was considered effective if the ratio was superior to 10. Then, ileum samples were diluted in the assay buffer to achieve a concentration of 1 mg/mL of proteins. Assay buffer was composed of Tris-HCl 10 mM (T15760, Sigma-Aldrich, St. Louis, MO, USA) and KCl 120 mM (P5405, Sigma-Aldrich, St. Louis, MO, USA). For the basal reading, 95 μL of assay buffer + 10 μL of 1 mg/mL sample were plated in triplicate. Subsequently, 20 μL of reduced Cytochrome *c* was added to initiate the reaction, which was monitored kinetically (for 15 min with readings at 30-s intervals for a total of 30 readings per well) using a microplate spectrophotometer Thermo Scientific Multiskan GO at a wavelength of 550 nm (Thermo Fisher Scientific, Waltham, MA, USA).

Enzyme activity was calculated by interpolating the values of the samples obtained in the linear part of the reaction kinetics, using the following formula:

Unit/mL = ΔA/min × dil × 0.125 mL

mL of enzyme × 21.84

△A/min → change in absorbance over time

dil → 1 mg/mL of protein

0.125 mL → volume of solution in each well

mL of enzyme → 10 μL = 0.01 mL

21.84 → molar extinction coefficient

Cytochrome *c* oxidase activity is expressed in mU/mg of protein.

### 4.8. Immunofluorescence of Intestinal Paneth Cells

Immunofluorescence studies were performed to detect defensin 1–3-positive Paneth cells in formalin-fixed, paraffin-embedded mouse ileal sections, as previously reported [50]. Briefly, sections were incubated with blocking reagent for non-specific binding and successively with rabbit polyclonal anti-defensin 1–3 ([1:200], code PA5-103122, Invitrogen, Waltham, MA, USA) primary antibody, diluted in phosphate-buffered saline overnight at 4 °C. The sections were then exposed to anti-rabbit Alexa Fluor 555 conjugated ([1:300], code A21430, Invitrogen, Carlsbad, CA, USA) and nuclear counterstaining with TO-PRO3 ([1:300], code T3605, Invitrogen, Carlsbad, CA, USA), as previously described [51]. Sections were examined under a Leica TCS SP8 confocal laser-scanning microscope (Leica Microsystems, Mannheim, Germany) and samples were scanned by means of a 63× oil lens, using a sequential scan procedure.

### 4.9. Statistical Analysis

Data were presented as mean ± standard error of the mean (S.E.M.) and analyzed by the GraphPad Prism 11.0 (GraphPad Prism, San Diego, CA, USA). Statistical significances were determined by one-way ANOVA followed by Tukey’s post hoc test. *p*-values lower than 0.05 were considered significant.

## 5. Conclusions

In conclusion, the administration of this novel food supplement exerted a protective effect in the DSS-induced post-colitis IBS-like model, mitigating the local immune response and restoring the integrity of the intestinal epithelial barrier, facilitating butyrate bioavailability within the colonic epithelium. This evidence suggests a possible use of this supplement as a useful approach to manage post-inflammatory IBS-associated intestinal mucosal and epithelial layer alterations.

## Data Availability

The authors declare that all data supporting the findings are contained in the paper.

## Supplementary Materials

The following supporting information can be downloaded at: https://www.mdpi.com/article/10.3390/ijms27156629/s1.
