# Supplementary material for: A Novel Mixture Containing Enterococcus lactis SF68® Restores Gut Barrier Integrity in a DSS-Induced Murine Model of Post-Colitis IBS-like Symptoms
Source: Int J Mol Sci. 2026 Jul 25;27(15):6629. doi: 10.3390/ijms27156629 (PMC13467298; doi:10.3390/ijms27156629)
Supplement: Supplementary file 1 [file ijms-27-06629-s001.zip › ijms-4301416-supplementary.pdf]

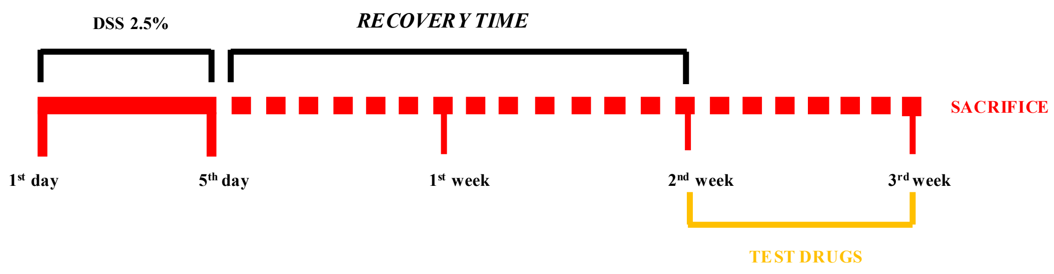

Supplementary figure S1. Schematic representation of dextran sulfate sodium-induced post colitis IBS-like model
